# Cold-responsive interaction between MdRAD23D1 and MdMYB15 confers cold stress tolerance via the CBF pathway in apple (*Malus domestica*)

**DOI:** 10.1371/journal.pgen.1012207

**Published:** 2026-06-25

**Authors:** Xiaoli Zhang, Benzhou Zhao, Xiaoyan Li, Hui Xia, Fengwang Ma, Dong Liang, Xiaoqing Gong

**Affiliations:** 1 College of Horticulture, Sichuan Agricultural University, Chengdu, China; 2 State Key Laboratory for Crop Stress Resistance and High-Efficiency Production/Shaanxi Key Laboratory of Apple, College of Horticulture, Northwest A&F University, Yangling, Shaanxi, China; Gregor Mendel Institute of Molecular Plant Biology, AUSTRIA

## Abstract

Low temperature is a major environmental factor that impairs plant growth and development, posing a significant threat to crop yield and quality. RAD23 (RADIATION SENSITIVE23) proteins belong to the UBL-UBA (Uiquitin-like-ubiquitin associated) family and function as shuttle factors in the UPS (ubiquitin proteasome system). Although UBL-UBA proteins are known regulators of plant stress responses, the function and mechanism of RAD23 in apple under cold stress are poorly understood. Here, we demonstrated that MdRAD23D1 is induced by 4 °C and positively regulates cold tolerance. Silencing *MdRAD23D1* impaired cold tolerance in both apple plants and calli. Conversely, its overexpression enhanced cold tolerance in transgenic tobacco, and apple calli and plants. We further demonstrated that MdRAD23D1 interacted with MdMYB15 protein via *in vivo* and *in vitro* assays. MdMYB15 functions as a negative regulator of cold stress tolerance. This is evidenced by the enhanced cold tolerance in apple calli and plants in which *MdMYB15* expression was silenced, contrasted with the reduced tolerance in materials of overexpressing *MdMYB15*. Furthermore, yeast one-hybrid (Y1H), dual-luciferase (Dual-LUC), and electrophoretic mobility shift assays (EMSA) showed that MdMYB15 could bind to the promoters of *CBF1*, *CBF2*, and *CBF3* and inhibit the expressions of the corresponding genes. In addition, MdRAD23D1 promoted MdMYB15 degradation under cold stress, thus enhancing the cold tolerance of apple. In summary, we proposed a mechanism for the response of apple to cold stress that is mediated by the ‘MdRAD23D1-MdMYB15-MdCBFs’ modula, which enhances our understanding of the regulation of cold tolerance in apple by UBL-UBA protein.

## Author summary

Low temperature is a major threat to apple production, limiting growth and reducing fruit quality. To survive cold stress challenges, plants have evolved sophisticated internal mechanisms. In this study, we discovered that apple plants naturally produce more MdRAD23D1 when exposed to low temperatures. This protein functions as a crucial defender; increasing its levels makes the plants more resilient to the cold, while removing it leaves them vulnerable. We found that MdRAD23D1 works by targeting a negative regulator named MdMYB15. Normally, MdMYB15 hinders the plant’s defense by blocking key cold-resistance genes. However, MdRAD23D1 promotes the degradation of MdMYB15, effectively removing this roadblock. This clearance allows the cold-resistance genes to activate, thereby strengthening the plant’s tolerance. Consequently, we identified a vital molecular module ‘MdRAD23D1-MdMYB15-MdCBFs’, which dictates how apples respond to cold. These findings deepen our understanding of plant biology and offer a promising strategy for breeding apple varieties that can better withstand harsh climates, securing future yields.

## 1. Introduction

Low temperature is a critical environmental factor that inhibits plant growth and development, limits crop yield, and reduces fruit quality. Cold stress includes chilling stress (0–15 °C) and freezing stress (<0 °C). Plants exhibit varying symptoms of chilling stress depending on their growth and developmental stages. For example, at the germination stage, chilling stress could cause delayed germination, reduced the germination rate, and weaken seedling growth. During the vegetative growth stage, chilling stress may induce symptoms such as plant etiolation, seedling yellowing, reduced photosynthetic efficiency, and decreased root vigor. In severe cases, it can lead to wilting or death. At the reproductive stage, chilling stress can result in phenotypes including poor-quality flower buds and reduced fruit set [1, 2]. Freezing stress induces the appearance of ice crystals in the interstitial spaces of plant cells and disrupts the physical structure of cell membranes, leading to a decrease in extracellular water potential. In severe cases, it results in cellular dehydration, a decrease in cold tolerance, and a decrease in the water content of plants [3, 4]. Therefore, improving plant cold tolerance is essential to increase agricultural productivity. One effective strategy is the development of transgenic plants with enhanced stress tolerance [5, 6].

Plants have developed sophisticated adaptive strategies in response to cold stress [7, 8]. CBFs/DREB1, which belonging to the AP2/ERF family, serve as central transcription factors in the cold stress response [9]. Following rapid induction by cold, they bind to *DRE*/*CRT cis*-elements and act as core regulators driving the expression of numerous cold-responsive genes, including RD29A, COR47, and KIN1 [10]. Following the initial discovery of three *CBF* genes (*CBF1*/*DREB1b*, *CBF2*/*DREB1c*, and *CBF3*/*DREB1a*) in *Arabidopsis*, CBF homologs have been cloned and identified in various other plants. For example, overexpression of *PpCBF1* in apple rootstock M26 significantly enhances cold tolerance [11]. Similarly, *VvCBF4* overexpression increases freezing resistance in grape [12]. To date, many TFs have been shown to regulate *CBF* expression. Key positive regulators include ICE1/2 (Inducer of CBF Expression 1/2) and CAMTAs (Calmodulin-binding transcription activators) [13, 14, 15], Conversely, several negative regulators, such as EIN3 (ethylene insensitive 3) [16], PIFs (phytochrome-interacting factors) [16] and MYB15 [17] have also been identified. Notably, SlMYB15 targeted by slymiR156e-3p positively regulates ABA-mediated cold tolerance in tomato [18]. MdMYB15L has been reported to negatively regulate anthocyanin accumulation and cold tolerance in apple calli [19], suggesting its potential role as a negative regulator in the cold signaling pathway.

Ubiquitination is a post-translational modification that contributes to stress responses in plants. In addition to the three critical enzymes E1 (ubiquitin-activating enzymes), E2 (ubiquitin-conjugating enzymes), and E3 (ubiquitin ligases) [20], the ubiquitin-proteasome system (UPS) also require a class of ubiquitin transporters to transport the ubiquitin-modified target protein to the 26S proteasome, which allows it to participate in ubiquitin degradation [21]. UBL-UBA (ubiquitin-like-ubiquitin-associated) proteins function as ubiquitin receptors and shuttles within the UPS. They typically contain an N-terminal UBL domain, which interacts with proteasome receptors such as Rpn1, Rpn10, and Rpn13, and a C-terminal UBA domain that binds to ubiquitinated target proteins. Additionally, many UBL-UBA proteins possess an STI (stress-inducible-1) domain [22, 23, 24]. When target proteins are tagged with polyubiquitin chains, UBL-UBA proteins recognize and deliver them to the 26S proteasome for degradation. Studies have shown that UBL-UBA proteins play a significant role in plant stress responses. For instance, the UBL-UBA protein OsDSK2a regulates gibberellin metabolism in rice, thereby mediating seedling growth and salt stress tolerance [25].

Another UBL-UBA protein, RAD23 (RADIATION SENSITIVE23), functions as a nucleotide excision repair factor in yeast (*Saccharomyces cerevisiae*), where it repairs UV-induced DNA damage [26]. In plants, RAD23 protein participates in various biological processes and stress responses. For example, in *Arabidopsis*, RAD23B is involved in degrading KRP1 (KIP-related protein 1) to regulate pollen development [27]. RAD23 also helps protect plants from ultraviolet radiation [28]. Additionally, following insect colonization, *Arabidopsis* RAD23C and RAD23D interact with the phytoplasma virulence effector SAP54 (secreted AY-WB protein), leading to degradation of MADS-box proteins and disruption of reproductive development [29]. Previously, we demonstrated that MdRAD23D1 mediates the drought response by regulating the degradation of the proline-rich protein MdPRP6 in apple (*Malus domestica*) [30]. We also showed that MdRAD23D1 affects water use efficiency (WUE) under long-term moderate drought stress in transgenic apple [31]. However, little is known about the role of *MdRAD23D1* in the responses of apple to cold stress.

Apple is a globally prominent fruit crop. Nevertheless, in several major production regions, cold stress significantly compromises both fruit quality and yield. In this study, we demonstrated that MdRAD23D1 positively regulates the response to cold stress by generating different transgenic materials. we also found that MdRAD23D1 could interact with MdMYB15 and promote its degradation via the UPS. MdMYB15 negatively regulated the cold stress response by directly binding to *MdCBF1/2/3* promoters and inhibiting their expression. Overall, these findings elucidate a new component of the apple cold stress regulatory network, laying the groundwork for the genetic enhancement of cold tolerance through molecular breeding.

## 2. Materials and methods

### 2.1. Plant materials and cold treatments

This study utilized the following plant materials: the tissue-cultured apple cultivar ‘GL-3’ (*Malus domestica*); the *Malus hupehensis* variety Pingyi Tiancha; ‘Orin’ apple (*Malus domestica*) calli cultures and tobacco (*Nicotiana nudicaulis* and *Nicotiana benthamiana*). The tissue-cultured wild-type (WT) ‘GL-3’ plants and three previously obtained *MdRAD23D1*-RNA interference (Ri) lines (using ‘GL-3’ as explants) were transferred to MS rooting medium to induce root development. The ‘GL-3’ apple plants with vigorous root growth were transferred to a mixture of organic substrate/vermiculite/perlite (3:1:1, v:v:v) for freezing treatment (-6 °C, 6 h). Before freezing treatment, the ‘GL-3’ and *MdRAD23D1*-Ri plants with the same growth status (approximately 15–20 cm tall) were randomly divided into three groups: 1) plants that were not treated with freezing (control group); 2) plants that were not treated in 4 °C for cold acclimatization (NA group); and 3) plants that were transferred to 4 °C for 12 h for cold acclimatization (CA group). The freezing treatments were performed as described in a previous study [32]. Both overexpression/Ri empty vector (OE-EV/OE-Ri) and corresponding transgenic Pingyi Tiancha seedlings were exposed to −6 °C for 8 h in a freezing treatment. One-month-old *Nicotiana nudicaulis* seedlings and transgenic lines (using *Nicotiana nudicaulia* as explants) were subjected to -5 °C for 6 h for freezing treatment. There were three biological replications and each biological replicate included 30 plants. To induce cold stress, 0.1 g of WT and transgenic apple calli (using ‘Orin’ as explants) were subjected to 4 °C for 25 d under continuous darkness. All plant materials were grown in a growth chamber at 23 °C, 16 h/8 h (light/dark) except for apple calli, which were grown under continuous darkness.

### 2.2. Vector construction and genetic transformation

The coding sequences (CDS) of *MdMYB15* were amplified from full-length complementary DNA (cDNA) of ‘Gold delicious’ apple (*Malus domestica*), and the sequences are shown in S1 Table. To obtain the overexpression vector, the CDSs of *MdMYB15* were introduced into the pCAMBIA2300 vector (with a GFP tag) and pGWB415 vector (with a HA tag) to obtain the *MdMYB15*-GFP and *MdMYB15*-HA vectors, respectively. The gene-specific fragment of *MdMYB15* was inserted into pK7GWIWG2D (with a GFP tag) to generate the *MdMYB15-*Ri vector. The CDS of *MdRAD23D1* was cloned into pCAMBIA2300 vector for genetic transformation of *Nicotiana nudicaulis.* All constructed vectors were introduced into *Agrobacterium tumefaciens* strain EHA105 using the heat-shock method. *Agrobacterium tumefaciens*-mediated genetic transformation of apple calli was performed following a previously described method [33]. The genetic transformation of *Nicotiana nudicaulis* was performed as described in a previous study [34]. The method of transient genetic transformation of Pingyi Tiancha was based on previous methods [35, 36]. Simply, the seedlings of Pingyi Tiancha with 4–6 true leaves were immersed in *Agrobacterium* suspension of OE-EV/*MdMYB15* or Ri-EV/Ri-*MdMYB15*, infiltrated under vacuum at 0.08 MPa for 20 min, blotted dry to remove excess bacterial solution, and then all seedlings were transferred to soil substrate for growth. After 3 d, positive plants were identified using RT-PCR and qRT-PCR. All primers are listed in S1 Table.

### 2.3. RNA extraction and gene expression analysis

The total RNA of apple leaves and calli was extracted using the Plant Total RNA Isolation Kit (Chengdu, Sichuan, China). First-strand cDNA synthesis was conducted using the PrimeScript RT Reagent Kit (TaKaRa, Shiga, Japan). A CFX96 Real-Time PCR System (Bio-Rad, CA, USA) was used for RT-qPCR. *MdMDH* and Ubiquitin were used as the reference gene to calculate the expression levels of target genes in apple and *Nicotiana nudicaulis*, respectively. All primers are listed in S1 Table.

### 2.4. Subcellular localization of MdMYB15

To analyze the subcellular localization of MdMYB15, empty GFP vector and MdMYB15-GFP fusion protein were transiently expressed in *Nicotiana benthamiana* epidermal cells as previously described methods [37]. After 60 h, tobacco leaves were collected, and fluorescence images were captured using a confocal microscope (Olympus FV1000, Tokyo, Japan).

### 2.5. Determination of physiological and biochemical indicators

Relative electrolyte leakage (REL) was measured using a DDS307A Ray Magnetic conductivity meter (Leici Instrument Co., Ltd., Shanghai, China) and calculated using the following formula: REL (%)= (S1-S0)/(S2-S0)*100% [38]. The content of malondialdehyde (MDA) was monitored using detection kits (Suzhou Comin Biotechnology Co., Ltd., Suzhou, China). Total chlorophyll was extracted with 80% acetone under continuous darkness as described in a previous study [30]. The *Fv*/*Fm* value was obtained using a three-dimensional chlorophyll fluorescence imaging system (FC800, PSI, Czech Republic). The fresh weight of apple calli was measured using balance scale. The survival rate of plants was counted using following formula: Survival rate (%)= number of surviving plants/total number of individuals plants*100%.

### 2.6. Y2H assay

The full-length CDS of *MdMYB15* was cloned into pGADT7 to generate the *MdMYB15*-AD fusion vector, and *MdRAD23D1-*BD was obtained in our previous study [30]. *MdRAD23D1*-BD and *MdMYB15*-AD were co-transformed into yeast strain Y2H Gold (Pyeast, Wuhan, China) and inoculated onto SD base/-Trp-Leu medium for 3 d. The transformants were transferred to SD base/-Trp-Leu-His-Ade + X-α-gal for another 3 d to test the interaction.

### 2.7. Split-LUC assay

The full-length CDS of *MdMYB15* was connected to the pRI-101-nLuc vector to generate the *MdMYB15*-nLuc fusion vector. *MdRAD23D1*-cLuc was obtained from our previous study [30]. *MdMYB15*-nLuc was introduced into *Agrobacterium tumefaciens* strain EHA105 by the heat-shock method. One-month-old *Nicotiana benthamiana* was used to inject specific combinations of *Agrobacterium* strain EHA105 instantaneously. After 48–60 h, the tobacco leaves were collected and luciferase expression signals were observed using an ultra-sensitive multifunctional imager (Uvitec, Cambridge, UK).

### 2.8. Pull-down assay

The full-length CDS of *MdMYB15* was inserted into pET28a to generate the *MdMYB15*-His fusion vector. The purified MdRAD23D1-GST protein was derived from our previous study [30]. After washing three times with 1 × TBS buffer, the anti-GST magnetic beads were incubated with 300 µL of MdRAD23D1-GST at 4 °C for 8–10 h with gentle shaking. After incubation, the mixture was washed three times with Tris-NaCl buffer, and 100 µL of MdMYB15-His protein was added for incubation at 23 °C for 2 h. Finally, the eluted proteins were immunoblotted using antibodies labeled with GST and His (Yeasen, Shanghai, China).

### 2.9. Co-IP assay

The specific combinations of *Agrobacterium* were transiently expressed in one-month-old *Nicotiana benthamiana* leaves. After 60 h, the tobacco leaves were collected and placed in liquid nitrogen for total protein extraction. Next, 20 µL of anti-GFP magnetic beads (Beyotime, Shanghai, China) and 500 µL of extracted protein were incubated at 4 °C for 8–10 h with gentle shaking. Finally, the eluted proteins were immunoblotted using antibodies labeled with GFP and HA (Yeasen, Shanghai, China).

### 2.10. Y1H assays

The promoter fragment of *MdCBFs* (-1500 bp) was connected to the pABAi vector to generate *proMdCBFs-*pABAi and digested by the BstBI enzyme. The *MdMYB15*-AD was introduced into the Y1H yeast strain with *proMdCBFs-*pABAi and inoculated into the SD base/-Ura-Leu medium according to the protocol of Clontech. The positive clone was transferred to SD base/-Ura-Leu medium with 200 ng/mL Aureobasidin (ABA) to test the interaction. Empty pGADT7 was used as a negative control.

### 2.11. Dual-LUC assay

The full-length CDS of *MdMYB15* and the promoter fragments of *MdCBFs* (-2000 bp) were cloned into the pGreenII 62-SK and pGreenII 0800-LUC vectors, respectively. The fusion vectors were introduced into *Agrobacterium tumefaciens* strain EHA105 by the heat-shock method. Next, specific combinations of *Agrobacterium* were injected into one-month-old leaves of *Nicotiana benthamiana.* After 60 h, luciferase activity was monitored using a Dual-luciferase detection kit (Yeasen, Shanghai, China), and the luciferase expression signals were observed using an ultra-sensitive multifunctional imager (Uvitec, Cambridge, UK).

### 2.12. EMSAs

The CDS of *MdMYB15* was cloned into the pMAL-c5X (labeled with MBP) vector to form *MdMYB15*-MBP. The recombined *MdMYB15*-MBP constructs were expressed in *Escherichia coli* BL21 (DE3). The MBP purification column was used to purify proteins (Qihai, Shanghai, China). The EMSAs were conducted using the EMSA kit (Beyotime, Shanghai, China). The probe sequences are listed in S1 Table.

### 2.13. Protein degradation assay

The total proteins of apple leaves and calli treated with cold stress were extracted using degradation buffer according to a previous study [39]. Next, 300 ng of extracted total proteins and 100 ng of purified MdMYB15-His protein were incubated at room temperature with gentle shaking. The reaction samples were collected at different times, and an anti-His antibody was used to detect the protein level of MdMYB15-His. For proteasome inhibitor treatments, 50 µM proteasome inhibitor MG132 was added 1 h before total protein extraction. For protein degradation *in vivo*, the different transgenic apple calli were collected at specific time points after treatment with 50 μM MG132 and 75 μM CHX. Next, total protein was extracted and anti-GFP antibody was used to monitor the protein level of MdMYB15-GFP.

### 2.14. Statistical analysis

All data were analyzed using IBM SPSS Statistics software (version20; SPSS Inc., Chicago, IL). One-way ANOVA, followed by Tukey’s multiple-range tests, and Student’s *t*-test were used to evaluate the significance of differences between groups (*P* < 0.05).

## 3. Results

### 3.1. MdRAD23D1 positively regulates cold tolerance in transgenic tobacco and apple

**Fig 1:**
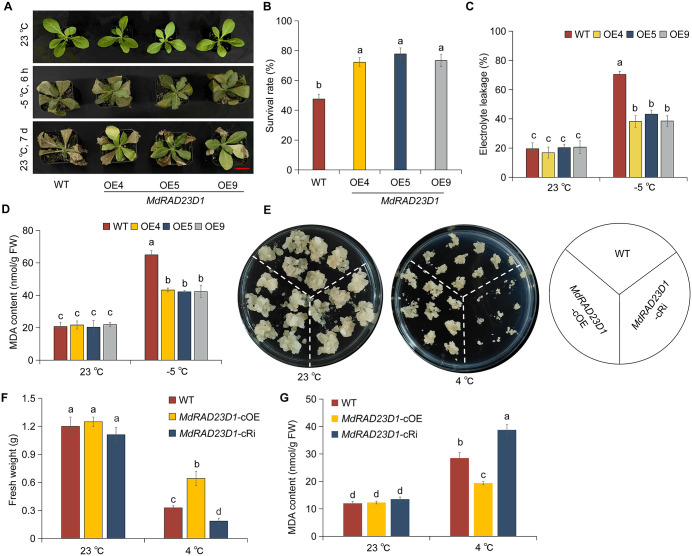
Role of *MdRAD23D1* in regulating cold tolerance of transgenic tobacco and apple calli. **(A)** The phenotypes of WT and *MdRAD23D1*-OE tobacco exposed to -5 °C for 6 h followed by 23 °C for 7 **d.** The scale bar = 5 cm. **(B-D)** The survival rate **(B)**, electrolyte leakage **(C)**, and MDA content (D) in WT and *MdRAD23D1*-OE tobacco under -5 °C for 6 **h. (E)** The phenotypes of WT and *MdRAD23D1*-cOE/Ri apple calli under 4 °C for 25 **d. (F-G)** The fresh weight (F) and MDA content (G) in WT and *MdRAD23D1*-cOE/Ri calli under 4 °C for 25 **d.** Data are means ± SD. Different letters indicate significant differences according to one-way ANOVA followed by Tukey’s multiple-range test (*P* < 0.05).

In previous study, we demonstrated that MdRAD23D1 positively regulated drought resistance by mediating the degradation of the proline-rich protein MdPRP6 [30]. In this study, the expression of *MdRAD23D1* was induced by 4 °C (S1A-S1B Fig). To investigate the biological function of *MdRAD23D1* in response to cold stress, we first obtained seven overexpressing transgenic tobacco lines and subjected the three lines with the highest expression levels (OE4, OE5, and OE9) to cold treatment (S2A-S2B Fig). Following 6 h freezing treatment at -5 °C, the leaves of WT plants completely lost their green pigmentation. In contrast, only the older leaves of *MdRAD23D1*-OE plants showed yellowish brown, while the younger leaves remained green. Following one week of recovery at room temperature, most leaves of the OE lines regained their green color, whereas in WT plants, nearly all leaves except the central young ones had withered (Fig 1A). The survival rate of OE lines was higher than that of WT plants (Fig 1B). Both electrolyte leakage and MDA content increased after freezing treatment, however, they remained lower in *MdRAD23D1*-OE plants than WT (Fig 1C-1D). We also employed previously obtained *MdRAD23D1*-cOE/Ri transgenic apple calli to test cold tolerance. After 4 °C treatment for 25 d, the *MdRAD23D1*-cOE calli were larger than WT, while the *MdRAD23D1*-cRi calli were the smallest (Fig 1E). Moreover, compared to the WT calli, *MdRAD23D1*-cOE calli exhibited higher fresh weight and lower MDA content, whereas *MdRAD23D1*-cRi calli showed the opposite trends under cold stress (Fig 1F-G).

**Fig 2:**
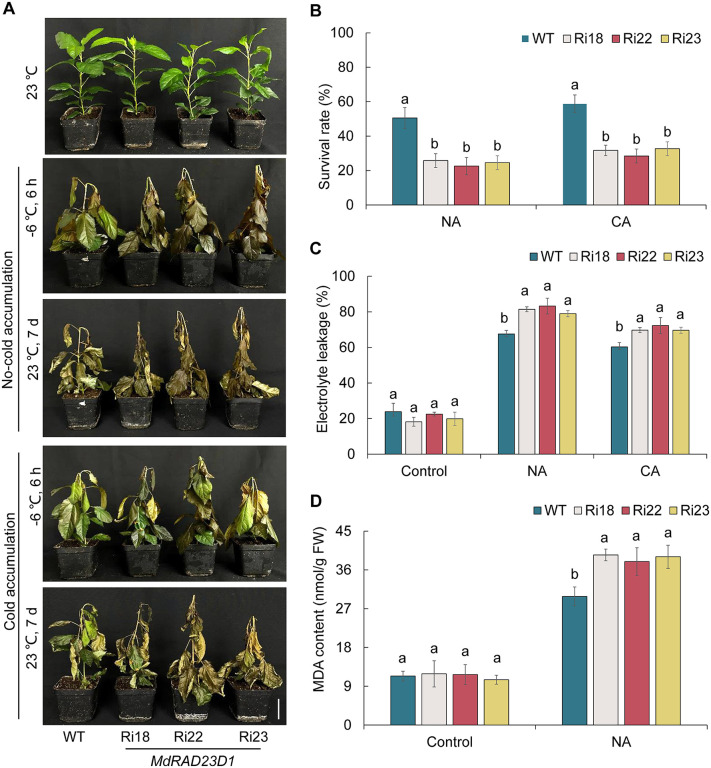
MdRAD23D1 positively regulates the cold tolerance of apple plants. **(A)** Morphological differences between WT and *MdRAD23D1*-Ri apple plants under 23 °C and -6 °C for 6 **h.** ‘GL-3’ and *MdRAD23D1*-Ri apple plants exposed to -6 °C for 6 h followed by 23 °C for 7 **d.** The scale bar = 5 cm. **(B-D)** The survival rate **(B)**, electrolyte leakage **(C)**, and MDA content (D) in WT and *MdRAD23D1*-Ri plants. Data are means ± SD. Different letters indicate significant differences between WT and *MdRAD23D1*-Ri plants exposed to the same treatment, according to one-way ANOVA followed by Tukey’s multiple range test (*P* < 0.05).

Three previously obtained *MdRAD23D1*-Ri apple lines (Ri18, Ri22, and Ri23) were used to further investigate the role of *MdRAD23D1* under low temperature stress [30]. No obvious phenotypic differences were observed under control conditions. However, after -6 °C for 6 h, most leaves of *MdRAD23D1-*Ri plants in the NA group (no cold acclimatization) withered, and only a small portion of WT leaves turned brown. A similar result was observed in the CA group (cold acclimatization), and *MdRAD23D1-*Ri plants showed more severe freezing stress symptoms and had fewer green leaves than WT plants. After recovery at 23 °C for one week, most leaves of the Ri lines withered in the CA group and even died in the NA group, while several leaves of WT plants remained alive and green (Fig 2A). In addition, the survival rate of *MdRAD23D1-*Ri plants was lower than that of WT plants in both NA and CA groups (Fig 2B). Conversely, the electrolyte leakage and MDA content were higher in Ri lines than in WT (Fig 2C-2D). Overall, these findings indicate that MdRAD23D1 plays a positive role in the cold stress response in plants.

### 3.2. MdRAD23D1 interacts with MdMYB15

**Fig 3:**
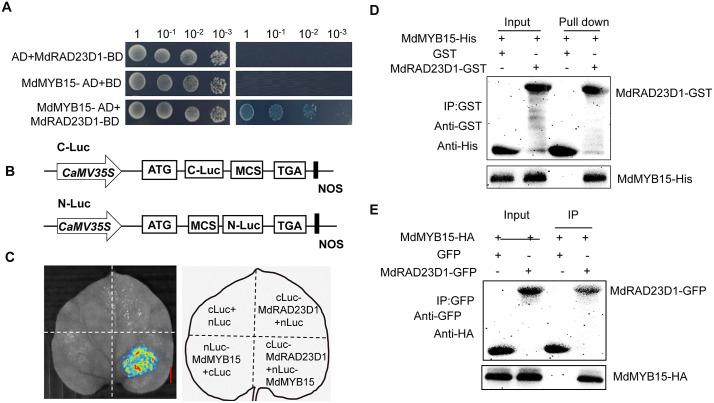
MdRAD23D1 interacts with MdMYB15 *in vivo* and *in vitro.* **(A)** Yeast two-hybrid (Y2H) assay. The CDSs of *MdRAD23D1* and *MdMYB15* were connected to the pGBKT7 and pGADT7 vectors, respectively. The positive clones were screened on SD-Trp/-Leu/-His/-Ade + X-α-gal medium. **(B)** The schematic diagram of the linked Split-Luc vectors. **(C)** The Split-Luc assay. The CDSs of *MdRAD23D1* and *MdMYB15* were connected to pRI-101-cLuc and pRI-101-nLuc to generate 35S::*MdRAD23D1*-cLuc and 35S::*MdMYB15*-nLuc, respectively. The scal bar = 0.8 cm. Different combinations of *Agrobacterium* were injected into 4-week-old *Nicotiana benthamiana* leaves, and the fluorescence signal was observed approximately 60 h after injection. **(D)** Pull-down assay. The MdMYB15-His protein was incubated with MdRAD23D1-GST. The eluted proteins were detected using anti-GST and anti-His antibodies. **(E)** Co-IP assay. The *MdRAD23D1*-GFP and *MdMYB15*-HA constructs were transiently expressed in *Nicotiana benthamiana* leaves. The total protein was extracted after 60 h, and the eluted mixture was detected using anti-GFP and anti-HA antibodies.

To further investigate the mechanism by which MdRAD23D1 mediates the cold stress response, we performed a Y2H screen of an apple cDNA library. Subsequently, the transcription factor MdMYB15 was identified as a candidate interacting protein. To further test the interaction between MdRAD23D1 and MdMYB15, we first cloned the full-length CDS of *MdMYB15* from the apple genome and ligated it to pGADT7 for a Y2H assay. When yeast strains were transformed with both *MdRAD23D1*-BK and *MdMYB15*-AD, a clear blue color developed on the SD base/-Trp, Leu, His, Ade + x-α-Gal medium (Fig 3A). Additionally, a strong luciferase signal was observed when *MdRAD23D1*-cLuc and *MdMYB15*-nLuc were co-injected into *Nicotiana benthamiana* (Fig 3B-3C). We also performed a pull-down assay and observed a clear bound band of MdMYB15-His in the lanes in which MdRAD23D1 and MdMYB15 were co-incubated (Fig 3D). In the Co-IP assay, MdMYB15-HA protein was detected in the MdRAD23D1-GFP and MdMYB15-HA combination by HA antibody (Fig 3E). These results suggest that MdRAD23D1 interacts with MdMYB15 *in vivo* and *in vitro*.

### 3.3. MdMYB15 negatively regulates cold tolerance in transgenic tobacco and apple

**Fig 4:**
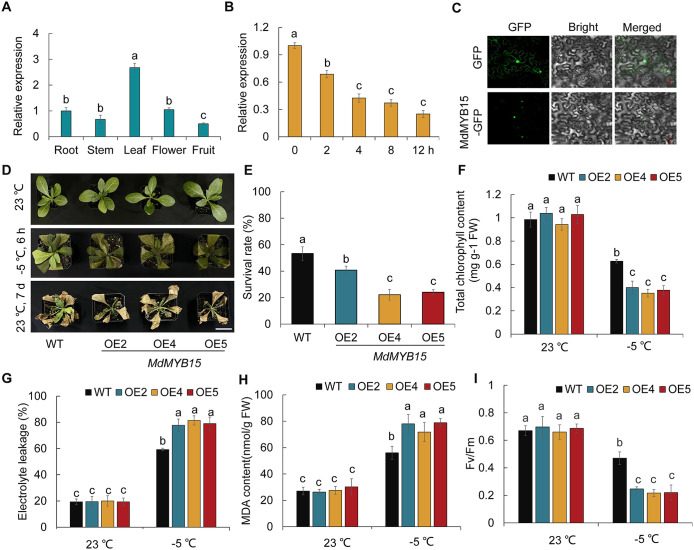
Functional characterization of *MdMYB15* in regulating cold tolerance of transgenic tobacco. **(A)** The expression of *MdMYB15* in the roots, stems, leaves, flowers, and fruits of apple according to qRT-PCR. **(B)** The expression of *MdMYB15* under cold stress according to qRT-PCR. The ‘GL-3’ plants were exposed to 4 °C for 12 h, and the leaves were collected for expression detection. **(C)** The subcellular localization of MdMYB15 in tobacco epidermal cells. The scale bar = 10 μm. Four-week-old *N. benthamiana* with good growth was selected for *Agrobacterium* injection. Empty GFP vector was used as a negative control. The fluorescence images were obtained by confocal microscopy (FV1000; Olympus, Tokyo, Japan). **(D)** Phenotypic differences between WT and *MdMYB15*-OE tobacco plants under freezing stress. The scale bar = 4 cm. **(E)** Survival rate of WT and *MdMYB15*-OE tobacco plants under -5 °C for 6 **h.** (F–I) The total chlorophyll content **(F)**, electrolyte leakage **(G)**, MDA content **(H)**, and *Fv/Fm* value (I) of WT and *MdMYB15*-OE tobacco under 23 °C and -5 °C conditions. Error bars indicate the SD of three biological replicates. Different letters indicate significant differences according to one-way ANOVA followed by Tukey’s multiple-range test (*P* < 0.05).

We first examined the expression pattern of *MdMYB15* in apple tissues and under cold stresses conditions. *MdMYB15* was expressed in roots, stems, leaves, flowers, and fruit, with the highest expression in leaves (Fig 4A). The expression of *MdMYB15* was continuously suppressed by cold stress (Fig 4B). Subcellular localization analysis revealed that MdMYB15 was localized to the nucleus (Fig 4C). To identify the function of *MdMYB15* under cold stress, we stably overexpressed *MdMYB15* in tobacco and three positive lines with higher *MdMYB15* expression (OE2, OE4, and OE5) were used for subsequent cold stress (S3A-S3B Fig). Following a 6 h freezing treatment at -5 °C, both WT and *MdMYB15*-OE plants exhibited cold damage. Notably, the young middle leaves of WT plants remained green, whereas those of OE plants turned mostly brown. After a 7 d recovery at 23 °C, all plants showed regreening in the middle leaves, but OE lines produced significantly fewer green leaves than the WT (Fig 4D). Consistently, the *MdMYB15*-OE lines also displayed a lower survival rate and reduced total chlorophyll content after freezing stress compared to WT (Fig 4E-4F). Furthermore, under cold stress, the electrolyte leakage in OE2, OE4, and OE5 lines was significantly elevated by 17%, 19% and 18%, respectively, compared to WT plants (Fig 4G). Although MDA content increased in all plants, it remained markedly higher in the *MdMYB15*-OE lines than in WT plants (Fig 4H). Conversely, the *MdMYB15*-OE plants exhibited a lower *Fv/Fm* than the WT under -5 °C conditions (Fig 4I).

**Fig 5:**
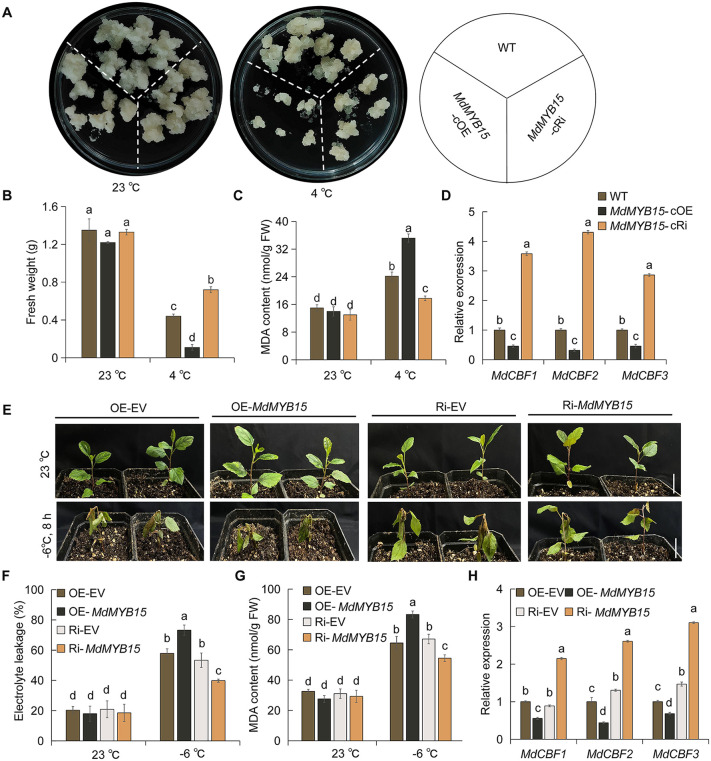
MdMYB15 negatively regulates the cold tolerance of apple calli and plants. **(A)** Growth phenotypes of WT and *MdMYB15*-cOE/Ri transgenic apple calli after 4 °C for 25 **d. (B-C)** Fresh weight (B) and MDA content (C) in **(A)**. **(D)** The relative expression level of *MdCBFs* in WT and *MdMYB15*-cOE/Ri transgenic apple calli under 4 °C conditions. **(E)** Morphological differences of empty control and *MdMYB15*-OE/Ri transgenic apple seedlings by transient transformation of leaves under 23 °C and -6 °C for 8 **h. (F–G)** Electrolyte leakage (F) and MDA content (G) in **(A)**. **(H)** The relative expression of *MdCBFs* in empty control and *MdMYB15*-OE/Ri transgenic apple seedlings under freezing conditions. Error bars indicate the SD of three biological replicates. Different letters indicate significant differences according to a one-way ANOVA followed by Tukey’s multiple-range test (*P* < 0.05).

To further investigate the role of MdMYB15 in regulating cold tolerance in apple, we obtained *MdMYB15*-cOE/Ri transgenic apple calli and tested their cold tolerance (S4A-S4D Fig). After 25 d at 4 °C, the *MdMYB15*-cOE calli were smaller, whereas the *MdMYB15*-cRi calli were larger than WT calli (Fig 5A). Compared to WT calli, the fresh weight of *MdMYB15*-cOE calli was significantly lower, while that of the *MdMYB15*-cRi calli was higher (Fig 5B). Under 4 °C treatment, the MDA content was lower in *MdMYB15*-cRi calli and higher in *MdMYB15*-cOE calli than in WT calli (Fig 5C). Furthermore, compared to the WT, the expression levels of *MdCBF1*, *MdCBF2* and *MdCBF3* were higher in *MdMYB15*-cRi calli and lower in *MdMYB15*-cOE calli under 4 °C stress (Fig 5D). We also generated *MdMYB15*-overexpressing/silenced seedlings by transient transformation of Pingyi Tiancha leaves (S5A-S5D Fig). All seedlings were treated with 4 °C for cold acclimatization and then transferred to -6 °C for 8 h. The *MdMYB15*-OE seedlings had more browned leaves compared with OE-EV seedlings; their leaves were also significantly wilted and more severely drooping. However, the cold tolerance of seedlings was enhanced when *MdMYB15* expression was inhibited (Fig 5E). The electrolyte leakage and MDA content were higher in *MdMYB15*-OE seedlings than in *MdMYB15*-Ri seedlings (Fig 5F-5G). The expression levels of *MdCBF1*, *MdCBF2*, and *MdCBF3* were higher in *MdMYB15*-Ri seedlings than in *MdMYB15*-OE seedlings (Fig 5H). These results suggest that *MdMYB15* negatively regulates cold tolerance by affecting the expression of *MdCBFs*.

### 3.4. MdMYB15 inhibits MdCBFs expression by binding to their promoters

**Fig 6:**
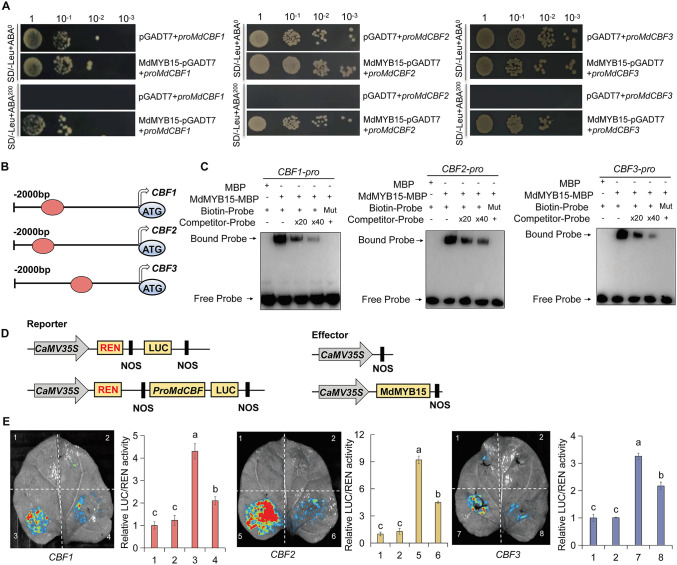
MdMYB15 directly binds to the promoters of *MdCBFs* and represses their expressions. **(A)** Y1H assay showed that MdMYB15 binds to the promoters of *MdCBF1/2/3*. **(B)** Diagram of the potential MdMYB15-binding sites within the *MdCBF* promoters. **(C)** MdMYB15-MBP could bind to the promoter regions of *MdCBFs* according to EMSAs. Arrows indicate protein-DNA complexes or free probes. **(D)** Schematic diagram of the constructed reporter and effector vectors. **(E)** Fluorescence observations and relative LUC/REN activity values in dual-LUC assays. Error bars indicate the SD of three biological replicates. Different letters indicate significant differences according to a one-way ANOVA followed by Tukey’s multiple-range test (*P* < 0.05). 1, empty reporter and effector; 2, empty reporter + 35S::MdMYB15; 3, *proMdCBF1*::LUC + empty effector; 4, *proMdCBF1*::LUC + 35S::MdMYB15; 5, *proMdCBF2*::LUC + empty effector; 6, *proMdCBF2*::LUC + 35S::MdMYB15; 7, *proMdCBF3*::LUC + empty effector; 8, *proMdCBF3*::LUC + 35S::MdMYB15.

Given that the expression of *MdCBFs* was inhibited in *MdMYB15*-OE plants but induced in *MdMYB15*-Ri under cold stress, we first employed a Y1H assay to detect whether MdMYB15 could bind to the promoters of *MdCBFs*. The Y1H strain was co-transformed with pGADT7/*MdMYB15*-pGADT7 and *proMdCBF1/2/3.* Compared with control, *MdMYB15*-pGADT7 and pro*MdCBF1/2/3* grew well on SD/-Leu medium supplemented with 200 mM Aureobasidin A (AbA) (Fig 6A). EMSAs showed that MdMYB15 could bind to the promoters of *MdCBF1/2/3* (Fig 6B-6C). Furthermore, the 2000 bp promoter of each *MdCBF* gene and the full-length CDS of *MdMYB15* were linked to the reporter vector and effector vector, respectively (Fig 6D). When co-expressing the two empty vectors or empty vector together with 35S::*MdMYB15*, no fluorescence signals were detected. However, a strong fluorescence signal was observed when co-expressing *ProMdCBFs*::LUC and the empty effector vectors, and a weaker signal was observed when co-expressing the *ProMdCBFs*::LUC and 35S::*MdMYB15* vectors (Fig 6E). Relative LUC/REN activities were detected, and lower values were observed when co-expressing the *ProMdCBFs*::LUC and 35S:*:MdMYB15* vectors compared with the *ProMdCBFs*::LUC and empty effector vector (Fig 6E). These results demonstrate that MdMYB15 inhibits the expression of *MdCBF1/2/3* by directly binding to their promoters.

### 3.5. MdRAD23D1 promotes MdMYB15 degradation under cold stress

**Fig 7:**
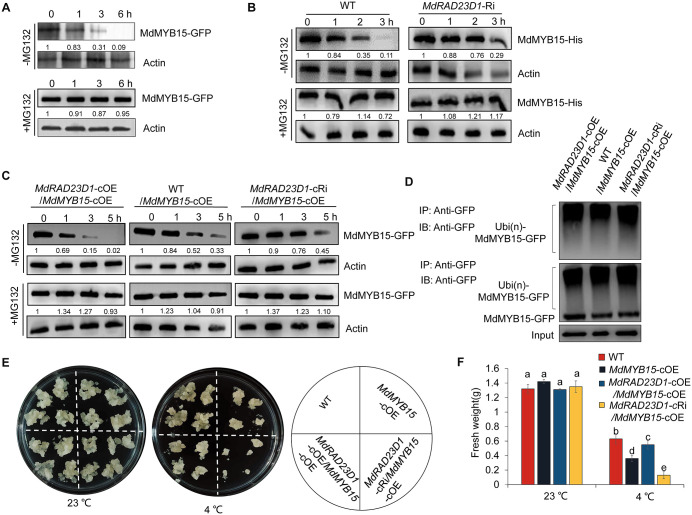
MdRAD23D1 promoted MdMYB15 degradation through the 26S proteasome pathway. **(A)** Effects of MG132 on MdMYB15 degradation. 25-d-old *MdMYB15*-GFP transgenic calli were treated with (+) or without (-) 50 µM MG132 for the indicated time. **(B)** Cell-free degradation assays of MdMYB15-His protein. The proteins of cold-treated WT and *MdRAD23D1*-Ri apple plants were extracted and incubated with MdMYB15-His protein for the indicated periods. The protein levels of MdMYB15-His were detected by immunoblotting using the anti-His antibody. **(C)** MdRAD23D1 promoted MdMYB15 degradation *in vivo*. The protein levels of *MdMYB15*-GFP in three types of transgenic calli were detected at specified time points (treated with CHX ± MG132) under cold stress. **(D)** Ubiquitination of MdMYB15 *in vivo*. The total proteins were separately extracted from the same three calli in (C) and immunoprecipitated using anti-GFP magnetic beads. (E) The growth phenotypes of WT and different types of transgenic apple calli after 4 °C for 25 d. (F) Fresh weight in (E). WT in (B) was ‘GL-3’ apple (*Malus domestica*); *MdRAD23D1*-cOE, transgenic apple plants overexpressing *MdRAD23D1*; *MdMYB15*-cOE, transgenic apple calli expressing 35S::*MdMYB15-GFP*. *MdRAD23D1*-cOE/*MdMYB15*-cOE, transgenic apple calli co-expressing 35S::*MdRAD23D1-HA* and 35S::*MdMYB15-GFP*. All the transgenic apple calli were generated using ‘Orin’ calli (*M. domestica*). Different letters indicate significant differences according to a one-way ANOVA followed by Tukey’s multiple-range test (*P* < 0.05) in (F).

Given that MdRAD23D1 functions as a ubiquitin receptor and shuttle protein, we hypothesized that it mediates the degradation of MdMYB15 by targeting it to the 26S proteasome. To test this, we pretreated *MdMYB15*-GFP transgenic calli with the proteasome inhibitor MG132 and detected protein levels using a GFP antibody. In the absence of MG132, the MdMYB15-GFP protein level decreased, whereas its degradation was effectively blocked when MG132 was present (Fig 7A). Furthermore, to assess MdMYB15 degradation in apple, we incubated purified MdMYB15-His protein with total protein extracts from cold-treated WT and *MdRAD23D1*-Ri apple leaves. The MdMYB15-His protein level decreased under cold stress in both WT and *MdRAD23D1*-Ri plants. However, this degradation was significantly attenuated in *MdRAD23D1*-Ri plants compared to WT plants (Fig 7B). To further assess the regulatory role of MdRAD23D1 on MdMYB15 stability, we monitored MdMYB15-GFP protein levels in different transgenic calli (S6A-S6D Fig). Total protein was extracted from *MdRAD23D1*-cOE/*MdMYB15*-cOE, *MdMYB15*-cOE, and *MdRAD23D1*-cRi/*MdMYB15*-cOE calli that had been treated at 4 °C and exposed to CHX for 10 h prior to collection. At each time point, MdMYB15-GFP degradation was markedly accelerated in *MdRAD23D1*-cOE/*MdMYB15*-cOE calli relative to *MdMYB15*-cOE controls, whereas it was significantly delayed in *MdRAD23D1*-cRi/*MdMYB15*-cOE calli. The degradation was effectively suppressed upon addition of MG132 (Fig 7C). Furthermore, the ubiquitination levels of MdMYB15-GFP in the co-transgenic calli was detected using anti-GFP and anti-Ubi antibodies and no significant change was observed in its ubiquitination levels corresponding to different *MdRAD23D1* levels (Fig 7D). The inhibitory effect of MdMYB15 on the cold stress response was alleviated by *MdRAD23D1* in transgenic calli under 4 °C, as reflected by the superior growth phenotype and greater fresh weight of *MdRAD23D1*-cOE/*MdMYB15*-cOE calli compared with *MdMYB15*-cOE calli (Fig 7E-7F). These results indicate that MdMYB15 can be degraded via UPS, and that MdRAD23D1 promotes its degradation, thereby affecting the cold resistance of apple.

## 4. Discussion

Cold stress restricts plant growth, development, and productivity. The cultivation of fruit trees depends largely on climatic conditions, with apple buds and being particularly susceptible to freezing damage [40, 41]. Therefore, screening cold-responsive genes and investigating their underlying stress resistance mechanisms are essential for breeding fruit trees with enhanced stress tolerance using biotechnology approaches.

The UBL-UBA protein functions as a transporter within the UPS system, delivering ubiquitination-labeled target proteins to the 26S proteasome for degradation [42]. RAD23 belongs to a family of UBL-UBA proteins and has been identified in various plants, including *Arabidopsis*, rice, and apple [22, 43, 44]. An increasing number of studies have focused on examining the function of *RAD23* in plant growth and development. For instance, *Arabidopsis* RAD23B can regulate pollen development by mediating the degradation of KRP1 [27]. Studies have also demonstrated that UBL-UBA proteins play important roles in plant stress response. For example, rice OsDSK2a modulates gibberellin metabolism by promoting the degradation of the substrate protein EUI, thereby regulating seedling growth and salt stress responses [25]. In our previous studies, we showed that MdRAD23D1 positively regulates drought stress responses by mediating the degradation of a proline-rich protein in apple [30]. We also found that MdRAD23D1 affects water use efficiency (WUE) under long-term moderate drought stress in transgenic apple [31]. Here, we investigated the function of *MdRAD23D1* in the cold stress response of apple and its underlying mechanism. The results showed that *MdRAD23D1* expression was up-regulated and positively regulated the response to cold stress. These conclusions were made based on the cold tolerance of *MdRAD23D1*-OE tobacco and apple transgenic materials and the sensitivity of *MdRAD23D1*-Ri apple lines to cold stress (Fig 1-2).

MYB transcription factors constitute one of the largest families of transcription factors in plants. Some are involved in the cold responses by interacting with other factors and regulating the expression of downstream target genes involved in cold stress pathways [45]. For example, MdMYB308L positively regulates cold stress by interacting with MdbHLH33 to promote the expression of *MdCBF2* and *MdDFR* in apple [46]. MdMYB88 and MdMYB124 increase cold hardiness through both *CBF*-dependent and *CBF*-independent pathways in apple [37]. DgMYB2 improves cold resistance by directly targeting *DgGPX1* in chrysanthemum [47]. In addition to its role in cold stress responses, MYB also plays roles in responses to other types of stresses, including drought and heat [48,49]. We identified the MYB transcription factor MdMYB15 via a Y2H assay using MdRAD23D1 as bait. The interaction of MdRAD23D1 and MdMYB15 was further verified by Split-Luc, Pull-down, and Co-IP assays (Fig 3).

An increasing number of studies have shown that MYB15 plays an important role in plant responses to cold stress. For example, the overexpression of *MdMYB15L* negatively regulates cold tolerance by suppressing the expression of *CBF2* in red-fleshed apple calli [19]. MdWRKY40 improves cold tolerance by blocking the expression of *MdMYB15L* in apple [50,51]. PUB25 and PUB26 promote plant freezing tolerance by mediating the degradation the cold-signaling negative regulator MYB15 [52]. However, tomato SlMYB15 functions as a positive regulator of the cold stress response through *CBFs* and the ABA-mediated pathway [42, 18]. In our study, overexpression of *MdMYB15* in tobacco and apple materials decreased cold tolerance, whereas silencing of *MdMYB15* improved the cold tolerance in apple seedlings. Significantly increased transcript levels of *CBF1*, *CBF2*, and *CBF3* were observed in *MdMYB15*-silenced calli and apple seedlings, while markedly decreased expression of *CBFs* was observed in *MdMYB15*-overexpressing lines (Fig 4, Fig 5). Thus, the transcription levels of *CBFs* were negatively correlated with that of *MdMYB15.* Based on these findings, we hypothesized that the cold-sensitive phenotype of *MdMYB15* is caused by its inhibition *CBFs* expression. Subsequent Y1H, and EMSA and dual-luciferase assays confirmed this hypothesis (Fig 6).

Most UBL-UBA proteins involved in plant growth, development and stress mediate the degradation of other factors [22, 52]. The ubiquitin shuttle function of MdRAD23D1, a UBL-UBA protein, was identified in our previous study [30]. In this study, we found that MdRAD23D1 plays a positive role in the cold stress response of apple, whereas MdMYB15 negatively regulates this response. Based on the stability of MdMYB15 and its regulation by cold stress (Fig 7A), we asked whether MdRAD23D1 could transport MdMYB15 to the 26S proteasome to participate in its degradation, thereby enhancing the cold tolerance of apple. *In vivo* and *in vitro* assays demonstrated that MdRAD23D1 promoted the ubiquitin-mediated degradation of MdMYB15 under cold stress, which in turn affected the cold stress tolerance of apple calli (Fig 7B-7F). Overall, we revealed a potential molecular mechanism by which MdRAD23D1 positively modulates cold tolerance in apple plants.

**Fig 8:**
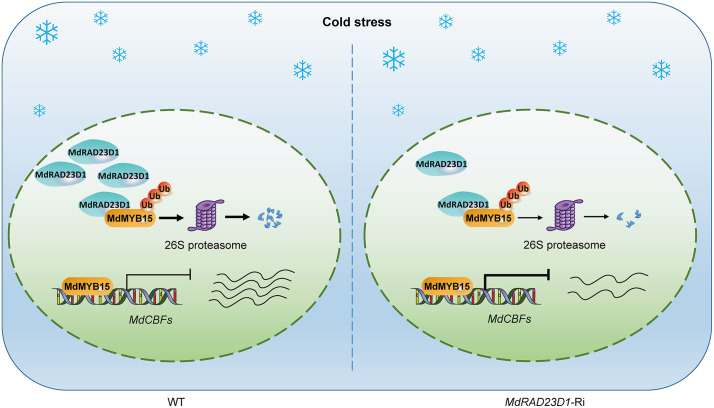
A model showing the role of MdRAD23D1 in the response of apple to cold stress. The expression of *MdRAD23D1* is induced by cold stress and plays a positive role in the response of apple to cold stress. MdMYB15 negatively regulates the response of apple to cold stress by inhibiting the transcription of *MdCBF1*, *MdCBF2*, and *MdCBF3*. MdRAD23D1 also interacts with MdMYB15 and promotes its degradation by the 26S proteasome pathway.

In summary, we propose a working model for how MdRAD23D1 regulates cold stress responses (Fig 8). Cold stress induces the expression of *MdRAD23D1*. MdRAD23D1 then interacts with the negative regulator MdMYB15, which represses the expression of *MdCBF* genes, and targets it for degradation. Through this mechanism, MdRAD23D1 alleviates MdMYB15-mediated repression, thereby enhancing the cold stress response in apple.

## Supporting information

S1 Data Raw data. (XLSX)

S1 Fig The expression detection of MdRAD23D1 under cold stress. (A) The transcription level of *MdRAD23D1* under 4 °C for 0, 2, 4, 6, 8 and 12 h. (B) The protein level of MdRAD23D1 under 4 °C for 0, 2, 4, 6, 8 and 12 h. ‘GL-3’ apple (*Malus domestica*) leaves exposed to 4 °C for 12 h were used to detect the expression level of MdRAD23D1 by qRT-PCR and west blotting assay.
(PDF)

S2 Fig Identification of *MdRAD23D1*-OE transgenic tobacco plants. (A) RT-PCR assay. P, the *MdRAD23D1*-pCAMBIA2300 vector. H_2_O, negative control. WT, wild type, here we used tobacco (*Nicotiana nudicaulis)*, which was also used as explants in generating transgenic tobacco plants. (B) RT-qPCR assay. Data are shown as the means ± SD. Different letters indicate significant differences according to one-way ANOVA followed by Tukey’s multiple range test (*P* < 0.05).
(PDF)

S3 Fig Identification of *MdMYB15* transgenic tobacco plants. (A) RT-PCR. WT, wild type, here we used tobacco (*Nicotiana nudicaulis)*, which was also used as explants in generating transgenic tobacco plants; P, the MdMYB15-pCAMBIA2300 vector. H_2_O, negative control. (B) RT-qPCR assay. Data are shown as the means ± SD. Different letters indicate significant differences according to one-way ANOVA followed by Tukey’s multiple range test (*P* < 0.05).
(PDF)

S4 Fig Identification of *MdMYB15* transgenic apple calli. (A-B) RT-PCR verification of *MdMYB15*-cOE and *MdMYB15*-cRi calli. (C-D) RT-qPCR detection of expression levels of *MdMYB15*-cOE and *MdMYB15*-cRi calli. P in (A), the recombined pCambia2300 vector expressed 35S::*MdMYB15-GFP*. P in (B), the recombined pK7GWIWG2D-MdMYB15 vector. WT, wild type, here we used ‘Orin’ apple calli (*Malus domestic*), which was also used as explants in generating transgenic apple calli. H_2_O, negative control. *MdMYB15*-cOE, transgenic apple calli expressed 35S::MdMYB15-GFP. *MdMYB15*-cRi, transgenic apple calli with suppressed expression of *MdMYB15* via RNA-interference. Asterisks indicate significant differences between WT and *MdMYB15*-cOE/Ri calli (*, *P* < 0.05).
(PDF)

S5 Fig Identification of *MdMYB15* transgenic Pingyi Tiancha seedlings. (A-B) RT-PCR verification of *MdMYB15*-OE and *MdMYB15*-Ri seedlings. (C-D) RT-qPCR detection of expression levels of *MdMYB15*-OE and *MdMYB15*-Ri seedlings. P in (A), the recombined pCambia2300 vector expressed 35S::*MdMYB15-GFP*. P in (B), the recombined pK7GWIWG2D-MdMYB15 vector. WT, wild type, here we used Pingyi Tiancha apple plants, which was also used as explants in generating transgenic apple seedlings. H_2_O, negative control. OE-EV, empty OE vector transferred to Pingyi tiancha seedlings. OE1–4, overexpression of *MdMYB15* in Pingyi tiancha seedings. Ri-EV, empty Ri vector transferred to Pingyi tiancha seedlings. Ri1–4, silencing of *MdMYB15* in Pingyi tiancha seedings. Data are shown as the means ± SD. Asterisks indicate significant differences between control and transgenic seedlings (*, *P* < 0.05).
(PDF)

S6 Fig Identification of different transgenic calli. (A) RT-PCR verification of *MdRAD23D1*-cOE/*MdMYB15*-cOE co-transgenic apple calli. P, the recombined pGWB415 vector expressed 35S::*MdRAD23D1-HA* (top), or the recombined pCambia2300 vector expressed 35S::*MdMYB15* (lower). (B) RT-PCR verification of *MdRAD23D1*-cRi/*MdMYB15*-cOE co-transgenic apple calli. P, the recombined pHellsgate2-*MdRAD23D1* vector (top), or the recombined pCambia2300 vector expressed 35S::*MdMYB15*-GFP (lower); (C) RT-qPCR analyses of the expression levels of *MdRAD23D1* and *MdMYB15* in *MdRAD23D1*-cOE/*MdMYB15*-cOE co-transgenic apple calli. (D) RT-qPCR verification of *MdRAD23D1*-cRi/*MdMYB15*-cOE co-transgenic apple calli. Data are shown as the means ± SD. Different lowercase letters above each bar indicate a statistically significant difference at *P* < 0.05.
(PDF)

S1 Table Application of primers and sequences. (XLSX)
