# Supplementary material for: Cold-responsive interaction between MdRAD23D1 and MdMYB15 confers cold stress tolerance via the CBF pathway in apple (Malus domestica)
Source: PLoS Genet. 2026 Jun 25;22(6):e1012207. doi: 10.1371/journal.pgen.1012207 (PMC13298947; doi:10.1371/journal.pgen.1012207)

S1 Fig. The expression detection of MdRAD23D1 under cold stress. (A) The transcription level of *MdRAD23D1* under 4 ℃ for 0, 2, 4, 6, 8 and 12 h. (B) The protein level of MdRAD23D1 under 4 ℃ for 0, 2, 4, 6, 8 and 12 h. 'GL-3' apple (*Malus domestica*) leaves exposed to 4 ℃ for 12 h were used to detect the expression level of MdRAD23D1 by qRT-PCR and west blotting assay.

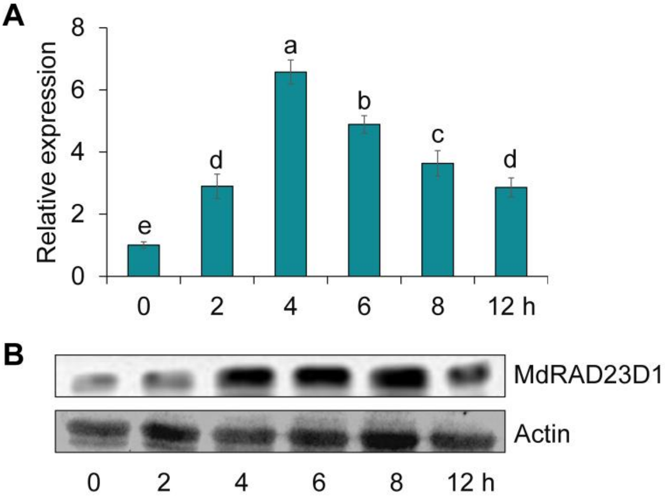

Supplement: S1 Fig — (A) The transcription level of MdRAD23D1 under 4 °C for 0, 2, 4, 6, 8 and 12 h. (B) The protein level of MdRAD23D1 under 4 °C for 0, 2, 4, 6, 8 and 12 h. ‘GL-3’ apple (Malus domestica) leaves exposed to 4 °C for 12 h were used to detect the expression level of MdRAD23D1 by qRT-PCR and west blotting assay. (PDF) [file pgen.1012207.s002.pdf]
