# Supplementary material for: Cold-responsive interaction between MdRAD23D1 and MdMYB15 confers cold stress tolerance via the CBF pathway in apple (Malus domestica)
Source: PLoS Genet. 2026 Jun 25;22(6):e1012207. doi: 10.1371/journal.pgen.1012207 (PMC13298947; doi:10.1371/journal.pgen.1012207)

S2 Fig. Identification of *MdRAD23D1*-OE transgenic tobacco plants. (A) RT-PCR assay. P, the *MdRAD23D1*-pCAMBIA2300 vector. H_2O, negative control. WT, wild type, here we used tobacco (*Nicotiana nudicaulis)*, which was also used as explants in generating transgenic tobacco plants. (B) RT-qPCR assay. Data are shown as the means ± SD. Different letters indicate significant differences according to one-way ANOVA followed by Tukey's multiple range test (*P* < 0.05).

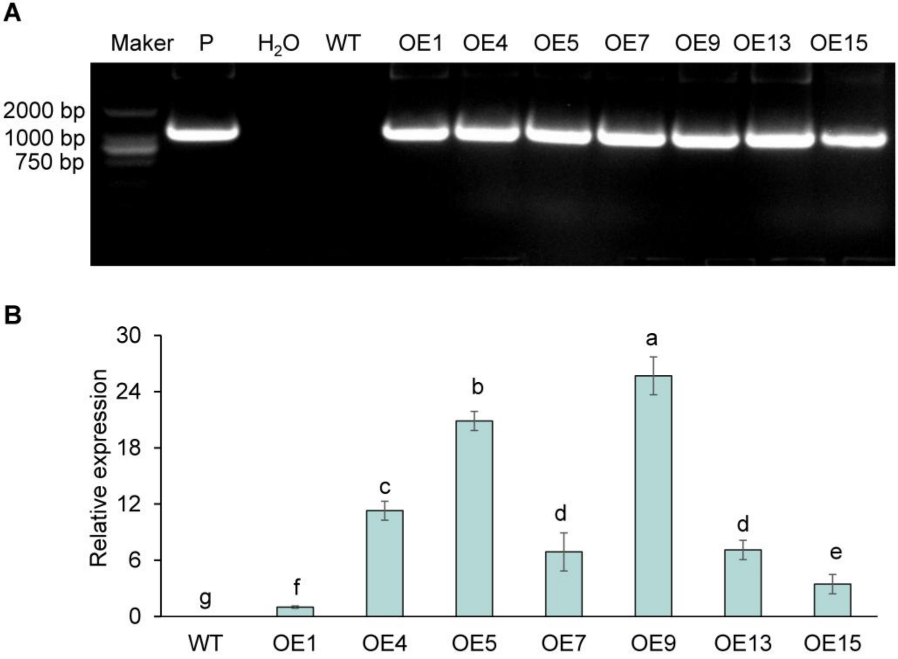

Supplement: S2 Fig — (A) RT-PCR assay. P, the MdRAD23D1-pCAMBIA2300 vector. H2O, negative control. WT, wild type, here we used tobacco (Nicotiana nudicaulis), which was also used as explants in generating transgenic tobacco plants. (B) RT-qPCR assay. Data are shown as the means ± SD. Different letters indicate significant differences according to one-way ANOVA followed by Tukey’s multiple range test (P < 0.05). (PDF) [file pgen.1012207.s003.pdf]
