# Supplementary material for: Cold-responsive interaction between MdRAD23D1 and MdMYB15 confers cold stress tolerance via the CBF pathway in apple (Malus domestica)
Source: PLoS Genet. 2026 Jun 25;22(6):e1012207. doi: 10.1371/journal.pgen.1012207 (PMC13298947; doi:10.1371/journal.pgen.1012207)

S3 Fig. Identification of *MdMYB15* transgenic tobacco plants. (A) RT-PCR. WT, wild type, here we used tobacco (*Nicotiana nudicaulis)*, which was also used as explants in generating transgenic tobacco plants; P, the MdMYB15-pCAMBIA2300 vector. H_2O, negative control. (B) RT-qPCR assay. Data are shown as the means ± SD. Different letters indicate significant differences according to one-way ANOVA followed by Tukey's multiple range test ($P < 0.05$).

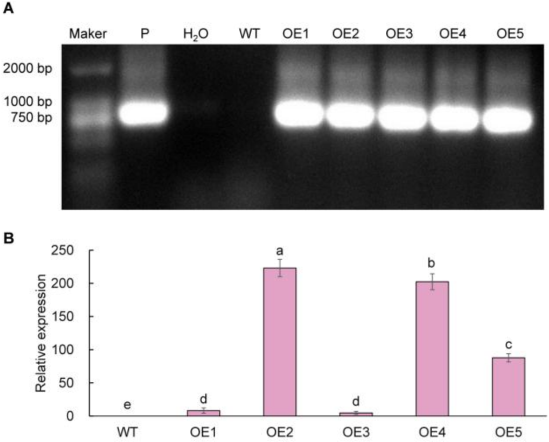

Supplement: S3 Fig — (A) RT-PCR. WT, wild type, here we used tobacco (Nicotiana nudicaulis), which was also used as explants in generating transgenic tobacco plants; P, the MdMYB15-pCAMBIA2300 vector. H2O, negative control. (B) RT-qPCR assay. Data are shown as the means ± SD. Different letters indicate significant differences according to one-way ANOVA followed by Tukey’s multiple range test (P < 0.05). (PDF) [file pgen.1012207.s004.pdf]
