# Supplementary material for: Cold-responsive interaction between MdRAD23D1 and MdMYB15 confers cold stress tolerance via the CBF pathway in apple (Malus domestica)
Source: PLoS Genet. 2026 Jun 25;22(6):e1012207. doi: 10.1371/journal.pgen.1012207 (PMC13298947; doi:10.1371/journal.pgen.1012207)

S4 Fig. Identification of *MdMYB15* transgenic apple calli. (A-B) RT-PCR verification of *MdMYB15*-cOE and *MdMYB15*-cRi calli. (C-D) RT-qPCR detection of expression levels of *MdMYB15*-cOE and *MdMYB15*-cRi calli. P in (A), the recombined pCambia2300 vector expressed 35S::*MdMYB15-GFP*. P in (B), the recombined pK7GWIWG2D-MdMYB15 vector. WT, wild type, here we used 'Orin' apple calli (*Malus domestic*), which was also used as explants in generating transgenic apple calli. H_2O, negative control. *MdMYB15*-cOE, transgenic apple calli expressed 35S::MdMYB15-GFP. *MdMYB15*-cRi, transgenic apple calli with suppressed expression of *MdMYB15* via RNA-interference. Asterisks indicate significant differences between WT and *MdMYB15*-cOE/Ri calli (*, $P< 0.05$).

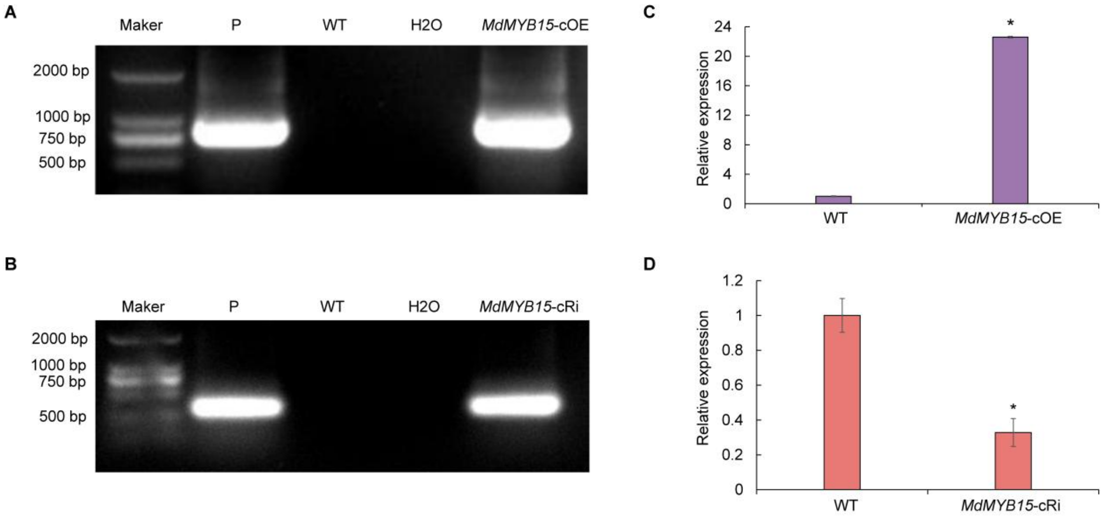

Supplement: S4 Fig — (A-B) RT-PCR verification of MdMYB15-cOE and MdMYB15-cRi calli. (C-D) RT-qPCR detection of expression levels of MdMYB15-cOE and MdMYB15-cRi calli. P in (A), the recombined pCambia2300 vector expressed 35S::MdMYB15-GFP. P in (B), the recombined pK7GWIWG2D-MdMYB15 vector. WT, wild type, here we used ‘Orin’ apple calli (Malus domestic), which was also used as explants in generating transgenic apple calli. H2O, negative control. MdMYB15-cOE, transgenic apple calli expressed 35S::MdMYB15-GFP. MdMYB15-cRi, transgenic apple calli with suppressed expression of MdMYB15 via RNA-interference. Asterisks indicate significant differences between WT and MdMYB15-cOE/Ri calli (*, P < 0.05). (PDF) [file pgen.1012207.s005.pdf]
