# Supplementary material for: Cold-responsive interaction between MdRAD23D1 and MdMYB15 confers cold stress tolerance via the CBF pathway in apple (Malus domestica)
Source: PLoS Genet. 2026 Jun 25;22(6):e1012207. doi: 10.1371/journal.pgen.1012207 (PMC13298947; doi:10.1371/journal.pgen.1012207)

S5 Fig. Identification of *MdMYB15* transgenic Pingyi Tiancha seedlings. (A-B) RT-PCR verification of *MdMYB15*-OE and *MdMYB15*-Ri seedlings. (C-D) RT-qPCR detection of expression levels of *MdMYB15*-OE and *MdMYB15*-Ri seedlings. P in (A), the recombined pCambia2300 vector expressed 35S::*MdMYB15-GFP*. P in (B), the recombined pK7GWIWG2D-MdMYB15 vector. WT, wild type, here we used Pingyi Tiancha apple plants, which was also used as explants in generating transgenic apple seedlings. H_2O, negative control. OE-EV, empty OE vector transferred to Pingyi tiancha seedlings. OE1-4, overexpression of *MdMYB15* in Pingyi tiancha seedings. Ri-EV, empty Ri vector transferred to Pingyi tiancha seedlings. Ri1-4, silencing of *MdMYB15* in Pingyi tiancha seedings. Data are shown as the means ± SD. Asterisks indicate significant differences between control and transgenic seedlings (*, $P< 0.05$).

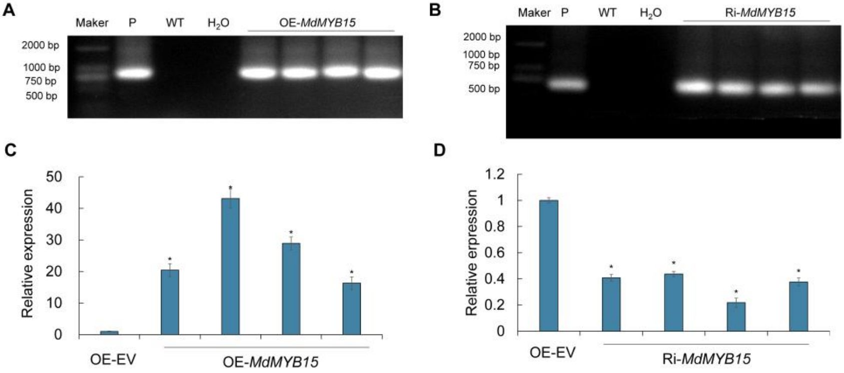

Supplement: S5 Fig — (A-B) RT-PCR verification of MdMYB15-OE and MdMYB15-Ri seedlings. (C-D) RT-qPCR detection of expression levels of MdMYB15-OE and MdMYB15-Ri seedlings. P in (A), the recombined pCambia2300 vector expressed 35S::MdMYB15-GFP. P in (B), the recombined pK7GWIWG2D-MdMYB15 vector. WT, wild type, here we used Pingyi Tiancha apple plants, which was also used as explants in generating transgenic apple seedlings. H2O, negative control. OE-EV, empty OE vector transferred to Pingyi tiancha seedlings. OE1–4, overexpression of MdMYB15 in Pingyi tiancha seedings. Ri-EV, empty Ri vector transferred to Pingyi tiancha seedlings. Ri1–4, silencing of MdMYB15 in Pingyi tiancha seedings. Data are shown as the means ± SD. Asterisks indicate significant differences between control and transgenic seedlings (*, P < 0.05). (PDF) [file pgen.1012207.s006.pdf]
