# Supplementary material for: Cold-responsive interaction between MdRAD23D1 and MdMYB15 confers cold stress tolerance via the CBF pathway in apple (Malus domestica)
Source: PLoS Genet. 2026 Jun 25;22(6):e1012207. doi: 10.1371/journal.pgen.1012207 (PMC13298947; doi:10.1371/journal.pgen.1012207)

S6 Fig. Identification of different transgenic calli.

(A) RT-PCR verification of *MdRAD23D1*-cOE/*MdMYB15*-cOE co-transgenic apple calli. P, the recombined pGWB415 vector expressed 35S::*MdRAD23D1-HA* (top), or the recombined pCambia2300 vector expressed 35S::*MdMYB15* (lower). (B) RT-PCR verification of *MdRAD23D1*-cRi/*MdMYB15*-cOE co-transgenic apple calli. P, the recombined pHellsgate2-*MdRAD23D1* vector (top), or the recombined pCambia2300 vector expressed 35S::*MdMYB15*-GFP (lower); (C) RT-qPCR analyses of the expression levels of *MdRAD23D1* and *MdMYB15* in *MdRAD23D1*-cOE/*MdMYB15*-cOE co-transgenic apple calli. (D) RT-qPCR verification of *MdRAD23D1*-cRi/*MdMYB15*-cOE co-transgenic apple calli. Data are shown as the means ± SD. Different lowercase letters above each bar indicate a statistically significant difference at $P<0.05$.

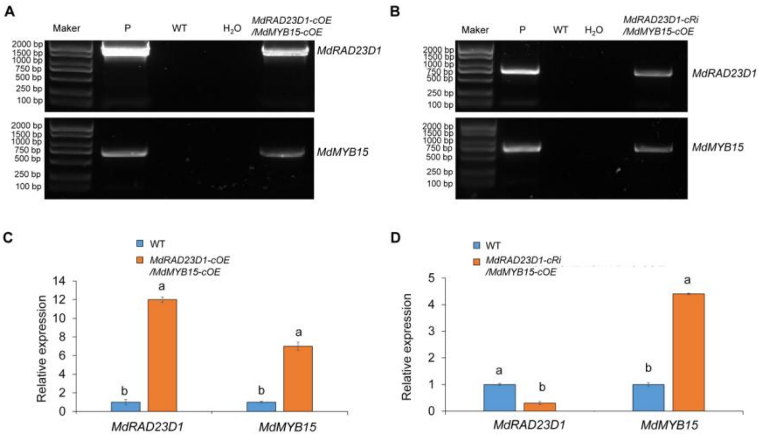

Supplement: S6 Fig — (A) RT-PCR verification of MdRAD23D1-cOE/MdMYB15-cOE co-transgenic apple calli. P, the recombined pGWB415 vector expressed 35S::MdRAD23D1-HA (top), or the recombined pCambia2300 vector expressed 35S::MdMYB15 (lower). (B) RT-PCR verification of MdRAD23D1-cRi/MdMYB15-cOE co-transgenic apple calli. P, the recombined pHellsgate2-MdRAD23D1 vector (top), or the recombined pCambia2300 vector expressed 35S::MdMYB15-GFP (lower); (C) RT-qPCR analyses of the expression levels of MdRAD23D1 and MdMYB15 in MdRAD23D1-cOE/MdMYB15-cOE co-transgenic apple calli. (D) RT-qPCR verification of MdRAD23D1-cRi/MdMYB15-cOE co-transgenic apple calli. Data are shown as the means ± SD. Different lowercase letters above each bar indicate a statistically significant difference at P < 0.05. (PDF) [file pgen.1012207.s007.pdf]
